# Plasmatic extracellular vesicle microRNAs in malignant pleural mesothelioma and asbestos-exposed subjects suggest a 2-miRNA signature as potential biomarker of disease

**DOI:** 10.1371/journal.pone.0176680

**Published:** 2017-05-04

**Authors:** Tommaso Cavalleri, Laura Angelici, Chiara Favero, Laura Dioni, Carolina Mensi, Claudia Bareggi, Alessandro Palleschi, Arianna Rimessi, Dario Consonni, Lorenzo Bordini, Aldo Todaro, Valentina Bollati, Angela Cecilia Pesatori

**Affiliations:** 1 Department of Gastroenterology, Humanitas Clinical and Research Center, Via Manzoni 56, Rozzano (Milan), Italy; 2 EPIGET—Epidemiology, Epigenetics and Toxicology Lab—Department of Clinical Sciences and Community Health, Università degli Studi di Milano, Via San Barnaba 8, Milan, Italy; 3 Fondazione IRCCS Ca’ Granda Ospedale Maggiore Policlinico, Department of Preventive Medicine, Epidemiology Unit, Via San Barnaba 8, Milan, Italy; 4 Fondazione IRCCS Ca’ Granda Ospedale Maggiore Policlinico, Medical Oncology Unit, Via F.Sforza 28, Milan, Italy; 5 Fondazione IRCCS Ca’ Granda Ospedale Maggiore Policlinico, Thoracic Surgery and Lung Transplantation Unit, Via F. Sforza 28, Milan, Italy; 6 Fondazione IRCCS Ca’ Granda Ospedale Maggiore Policlinico, Department of Preventive Medicine, Occupational Health Unit, Via San Barnaba 8, Milan, Italy; University of Valencia, SPAIN

## Abstract

**Background:**

Malignant Pleural Mesothelioma (MPM) is an aggressive cancer mainly caused by asbestos exposure and refractory to current therapies. Specific diagnostic markers for early MPM diagnosis are needed. Changes in miRNA expression have been implicated in several diseases and cancers, including MPM. We examined if a specific miRNA signature in plasmatic extracellular vesicles (EV) may help to discriminate between malignant pleural mesothelioma patients (MPM) and subjects with Past Asbestos Exposure (PAE).

**Methodology/Principal findings:**

We investigated 23 MPM patients and 19 cancer-free subjects with past asbestos exposure (PAE). We screened 754 miRNAs in plasmatic EVs by OpenArray and found 55 differential miRNAs using logistic regression models adjusted for age, sex, BMI, and smoking. Among the top-20 differential miRNAs chosen for validation by Real time PCR, 16 were confirmed. Using receiver operating characteristic (ROC) curve analysis, the most discriminating miRNA combination was given by miR-103a-3p + miR-30e-3p, which generated an AUC of 0.942 (95% CI 0.87–1.00), with a sensitivity of 95.5% and a specificity of 80.0%. Using multivariate Cox regression analysis including gender, age, BMI and smoking we found a Hazard Ratio for miR-103a-3p above the median of 0.37 (95%CI 0.13–1.13) and of 0.51 (95%CI 0.17–1.52) for miR-30e-3p.

**Conclusions:**

This study suggests EV-associated miR-103a-3p and miR-30e-3p are able to discriminate MPM from PAE subjects. Larger and prospective studies are needed to confirm these two-miRNA signature alone or in combination with other biomarkers as diagnostic tools for MPM.

## Introduction

Malignant Pleural Mesothelioma (MPM) is an aggressive cancer refractory to current therapies, with an incidence closely reflecting past asbestos exposure [1]. Recent projections have suggested that MPM incidence/mortality rates will continue to increase in the next 5 years in most European countries [2, 3]. The association between asbestos exposure and MPM is well established, with a mean latency time comprised between 30 and more than 40 years [4].

MPM is often diagnosed at late stages with poor prognosis. The development of minimal invasive test for early diagnosis would be of paramount clinical importance and would help in improving prognosis. Many studies have explored the possible role of MiRNAs in MPM tumorigenesis. MiRNAs are small, endogenous, single stranded noncoding RNAs of 20–22 nucleotides [5] that post-transcriptionally regulate gene expression by either triggering mRNA cleavage or repressing translation [6]. One single miRNA can regulate hundreds of mRNAs in interrelated gene pathways and a single mRNA can be targeted by several different miRNAs [7]. Changes in the expression of several miRNAs have been described in disease mechanisms that may be related to asbestos exposure such as oxidative stress [8] and regulation of inflammation [9].

The possible role of miRNAs as diagnostic or prognostic markers of MPM has been investigated in different studies [10–12]. However, most of them analysed miRNAs in tissue samples (neoplastic *vs* normal). Only few studies examined circulating miRNAs in serum or plasma (easily accessible markers) [13–17], but those investigations have been done only on a few patients and/or for a few candidate miRNAs, and results need confirmation.

In the present study, we investigated the specific signature of miRNAs, which are the cargo of extracellular vesicles (EV). The stability of EV-encapsulated miRNAs and the ease by which miRNAs can be detected in a quantitative manner make them an optimal biomarker for non-invasive diagnosis of diseases [18]. This is true also for free circulating miRNAs but, in addition, EVs show a more interesting functional meaning, as one of the well-described functions of EVs is to promote communication between the cells from which they are derived and their surrounding environment. It is then possible to speculate that the signature we could identify might be representative of the active crosstalk between cancer and the immune system, rather than a passive result of miRNA accumulating in plasma as a waste product [19].

We explored EV-associated miRNA expression profiles in MPM cases and subjects with past asbestos exposure (PAE), using Openarray. Differential miRNAs were further validated by Real time PCR. We present evidence of a two-miRNA signature that might help to discriminate between MPM and subjects with PAE.

## Methods

### Study population

The study population includes:

1. 23 MPM patients recruited at the Thoracic Surgery Unit, Fondazione IRCCS Ca’ Granda Ospedale Maggiore Policlinico, Milan, Italy, between October 2013 and August 2014. Pathological diagnosis was performed on pleural biopsies collected during video-assisted thoracoscopy surgery (VATS).
2. 19 subjects with a documented past occupational exposure to asbestos, which underwent a clinical surveillance program, in the same study period, at the Occupational Health Unit, Fondazione IRCCS Ca’ Granda Ospedale Maggiore Policlinico, Milan, Italy, as established by the Italian Law Dlgs 81/2008.

Individual written informed consent was collected from each participant before enrolment in compliance with the Ethics Committee of the “Ospedale Maggiore Policlinico” which approved the study (approval number 2423). MPM patients were followed-up to January 2016 to ascertain vital status.

### Asbestos exposure assessment

Information on lifetime asbestos exposure, in both occupational and environmental settings, has been collected through a standardized questionnaire administered to each subjects by trained interviewers. The questionnaire has been designed by the National Mesothelioma Register (ReNaM) and has been used for a long time for the ascertainment of asbestos exposure. Based on standardized criteria asbestos exposure is classified as occupational (definite, probable, possible), domestic, environmental, unlikely or unknown [20]. Demographic, lifestyle and smoking information was also collected.

### Blood collection, extracellular vesicles isolation and miRNA extraction

Each study participant was asked to donate a 7.5 ml blood sample, collected in EDTA Vacutainer tubes (Becton Dickinson, New Jersey, USA) and processed within 3 hours from blood drawing. None of the MPM patients underwent surgery, chemo or radiotherapy before blood collection. Blood was centrifuged at 400g for 15min to separate the plasma fraction from the blood cells. Plasma samples were centrifuged three times at increasing speeds (1000g, 2000g, 3000g) for 15min at 4°C to remove cell debris and aggregates. Supernatants were ultracentrifuged at 110 000g for 2h at 4°C. Simultaneously to study sample processing, we performed quality controls to assess the integrity and quality of the purified EVs. An example of quality control is reported in S1 Fig and includes: Transmission Electron Microscopy (TEM) analysis (panel A), Flow cytometry (panel B) and Nanosight analysis (panel C).

Enriched miRNAs were isolated with the miRNeasy purification kit (Qiagen Hilden, Germany) following the manufacturer's instructions. A final elution volume of 25 ul was used. To assess quality of miRNAs purification, random samples were analysed by 2100 Bioanalyzer (Agilent Technologies, Santa Clara, CA) using Agilent RNA 6000 Pico Kit. Isolated miRNAs were concentrated with Concentrator Plus (Eppendorf, Hamburg, GER) to 6,7 μl and stored at -80°C until further use.

### miRNA screening by OpenArray^®^ system

QuantStudio 12K Flex OpenArray Real-Time PCR System (Thermo Fisher Scientific, Waltham, MA, USA) was used to assess EV-associated miRNA profiling. The OpenArray reverse transcription reaction was performed, according to the manufacturer’s protocol, using 3 μl of miRNA in a 4.5 *μ*l mix of 0.75 *μ*l Megaplex™ RT Primers Pool A v2.1 and Pool B v3.0 (Life Technologies, Foster City, CA), 0.15 μl of dNTPs, 0.75 μl 10X RT Buffer, 0.90 μl of MgCl2 (25 mM), 0.1 μl of RNases Inhibitor (20 U/μl) and 1.5 μl of MultiScribe™ Reverse Transcriptase (50 U/μl). Reverse transcription reaction was performed using the following cycle conditions: 40 cycles at 16°C for 2 min, 42°C for 1 min and 50°C for 1 s, plus one cycle at 85°C for 5 min and final stage at 4°C.

To increase the ability to detect low abundance transcripts, a pre-amplification step was performed according to the manufacturer’s recommendation (“Low Sample Input Protocol (LSI) for profiling human miRNA using OpenArray® Platform”—Application Note 2011- Life Technologies). 7.5 *μ*l RT product was mixed with 1X Megaplex PreAmp primers (10X Human Pool A and B Cat. No. 4444748, Applied Biosystems), 1X TaqMan PreAmp master mix (2X, Cat No. 4391128, Applied Biosystems) to a final volume of 40 *μ*l. Pre-amplification reaction was performed using the following cycle conditions: 95°C for 10 min, 55°C 2 min, 72°C for 2 min, 16 cycles of 95°C for 15 s and 60°C for 4 min, than 99.9°C for 10 min to inactivate polymerases and final stage at 4°C. PreAmp product was first diluted with nuclease-free water to a ratio of 1:20, and TaqMan Open Array^®^ Real Time PCR Master Mix (2X) was added in ratio volume 1:1 (MasterMix/cDNA diluted). 7 μl reaction RT-PCR mix was aliquoted with robot MicroLab STAR Let (Hamilton Robotics, Birmingham, UK) in 8 wells of a 384-wells OpenArray^®^ plate. The manufacturer’s protocol was followed and the OpenArray panels were automatically loaded by the QuantStudio™AccuFill System Robot (Life Technologies, Foster City, CA).

Each panel enables the quantification of miRNA expression in 3 samples and up to 4 panels can be cycled simultaneously, allowing for the analysis of 12 samples on a QuantStudio 12K Flex Real-Time PCR system. 754 human miRNAs were amplified in each sample together with 16 replicates each of 4 internal controls (ath-miR159a, RNU48, RNU44 and U6 rRNA). The mean variation coefficient was 2.456 (Individual values are reported in S1 Table).

### miRNA normalization and screening data analysis

We obtained 758 Crt values (754 unique miRNAs and 4 internal controls) for each subject and an AmpScore value for each amplification curve. This value represents a quality measurement indicating a low signal in the amplification curve linear phase, with a range between 0 and 2.

**Fig 1:**
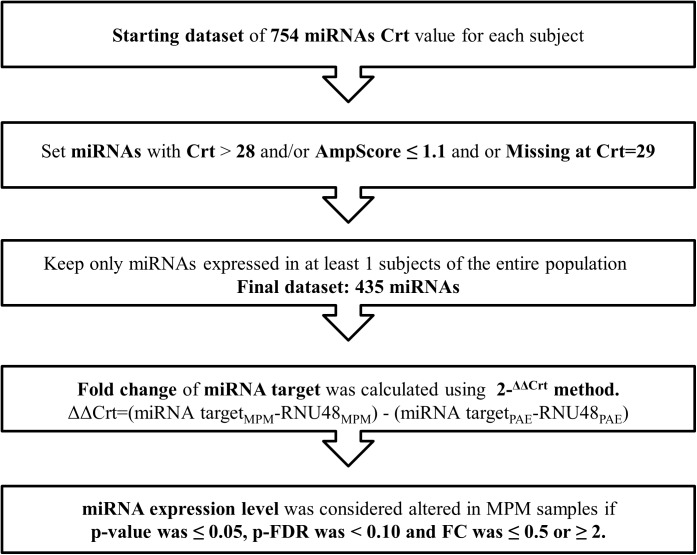
Data processing workflow.

MiRNAs with a Crt value>28 or AmpScore <1.1 or missing were considered unamplified and Crt value was set to 29 (Fig 1).

MiRNAs not amplified in at least one subject (n = 319) were excluded, resulting in 435 miRNAs being included in the analysis. Normalization was performed using RNU48. Additional normalization was performed using the average of the four miRNAs with the lowest standard deviation among subjects (miR-99a, miR-638, miR-720 and miR-1274a) obtaining similar results. miRNA expression was determined using the relative quantification 2-^ΔΔCrt^ [21].

### miRNA validation by Custom Taqman™ Low Density Array

Quantification of differentially expressed miRNAs was determined by Custom TaqMan™ Low Density Array (TLDA). Each miRNA was analysed in triplicate and RNU48 was used for data normalization. Reverse transcription of miRNAs was performed using Custom RT primer Pool (provided with Custom TaqMan™ Low Density Array). The RT reaction mix of 12 μL included: 3 μL of miRNAs, 6 μL Custom RT primer Pool, 0.3 μL dNTPs (100 nM), 3 μL MultiScribe™ Reverse Transcriptase (50 U/μL), 1.5 μL RT Buffer (10X), 0.19 μL RNase inhibitor (20 U/μL) and 1.01 μL nuclease-free water. RT was performed using a C1000 Thermal Cycler (Bio-Rad, Hercules, CA). The cDNAs were pre-amplified with a Custom Preamp Pool performed by manufacturer (Life Technologies), and each pre-amplification product was diluted 1:8. Custom TLDA were run in a 7900HT Fast real Time PCR System (Life Technologies, Foster City, CA), according to the manufacturer’s protocol. MiRNAs expression was calculated by the comparative cycle threshold (ΔCT) method and analysed with SDS Software (Life Technologies, Foster City, CA). The mean variation coefficient was 0.698 (Individual values are reported in S2 Table).

### Statistical analysis

Descriptive statistics were performed on all variables, reporting when normally distributed, means ± SD or frequencies, as appropriate. We applied χ^2^ for categorical variables and two sample t-test for continuous variables.

The mean value for each miRNA was calculated separately for MPM and PAE and their ratio was used to obtain the Fold Change (FC). For each miRNA a logistic regression model adjusted for age, sex, BMI and smoking habits was run to assess miRNA discrimination between MPM and PAE.

A Volcano plot was produced to select miRNAs characterized by more than ± 2-fold case-control differences (Fold Change >2 or < 0.5) with a p-value < 0.05 from logistic regression models.

The p-values were then adjusted for multiple testing by controlling the false discovery rate (FDR) according to the method of Benjamini and Hochberg [22]. After ordering by p-FDR, the top 20 differentially expressed miRNAs were selected for validation. A miRNA was considered to be differentially expressed if the p-value was ≤ 0.05, p-FDR was < 0.1 and FC was ≤ 0.5 or ≥2.

We used receiver-operating characteristics (ROC) curve calculated after logistic regression models adjusted for age, sex, BMI and smoking habits to evaluate the diagnostic ability of the validated miRNAs. Area under the Curve (AUC), Sensitivity (Se), Specificity (Sp) and corresponding 95% Confidence Intervals (CI) are reported. We also calculated Akaike and Bayesian Information Criteria (AIC and BIC) to corroborate miRNA ranking and model selection.

To investigate the potential association between miRNA expression and years of exposure (as a proxy of cumulative dose) we performed a linear regression model adjusted for age, sex, BMI and smoking habits.

Kaplan-Meier survival curves and log-rank tests were calculated stratifying MPM by miRNA expression below (= 0) or above (= 1) the median. To evaluate the independent prognostic value of miRNA on mortality we calculated hazard ratios (HR) with Cox multivariable regression models adjusted for age, sex, BMI and smoking. To examine the possible combination of two or more miRNAs, a score was built as sum of the index value (0,1) for each miRNA. The assumption of proportional hazard was checked with the log[-log(survival)] plot and by the time-dependent covariate test. We also used the Martingale residuals plot to evaluate whether a specific time-independent continuous covariate could be entered directly into the model, or if a transformation was necessary. All analyses were performed using SAS 9.3 (SAS Institute, Cary, NC).

## Results

### Characteristics of study participants

The main characteristics of the study participants are presented in Table 1.

**Table 1:** Main characteristics of the study population.

Variable	MPM (23)		PAE (19)	
**Sex,** n (%)				
Males	17 (73.9%)		15 (78.9%)	
Females	6 (26.1%)		4(21.1%)	
**Age,** (years), mean ± SD	70.2 ±7.8		66.5 ±6.4	
**BMI,** (Kg/m^2^), mean ± SD	25.1 ±4.4		26.3 ±4.2	
**Smoking,** n°(%),				
Current	5 (21.8)		3 (15.8)	
Never	6 (26.1)		9 (47.4)	
Former	12 (52.1)		7 (36.8)	
**Asbestos exposure,** n°(%),				
Definite Occupational	12 (52.3)		19 (100)	
Possible Occupational	2 (4.3)		-	
Environmental	2 (4.3)		-	
Unknown	7 (30.4)		-	
**Years of Exposure,** mean ± SD	21.8 ± 17.1		26.2 ± 12.4	
**Years of Latency,** mean ± SD	53.2 ± 11.7		46.3 ± 7.6	
**Years since Last Exposure,** mean ± SD	29.3 ± 20.5		20.1 ± 12.4	
**Histological subtype**				
Epithelioid	10			
Biphasic	11			
Sarcomatoid	1			
Not specified	1			

Most subjects in each group were males. BMI, age, and smoking distributions did not differ (p = 0.2; p = 0.81; p = 0.82) between the two groups. Among MPM, the most frequent histological types were biphasic (11) and epithelioid (10). Asbestos exposure was ascertained in 69.6% of MPM cases.

16 out of 19 PAE subjects had pleural plaques (a well-known indicator of past exposure to asbestos) at chest x-ray. In addition, two subjects had a radiological diagnosis of asbestosis. Duration of exposure and time since last exposure did not differ between the two groups (p = 0.3 and 0.09 respectively), whereas MPM patients showed a slightly longer latency (years since first exposure to diagnosis for MPM or blood collection for PAE subjects; p = 0.03).

### Deregulated miRNA

**Fig 2:**
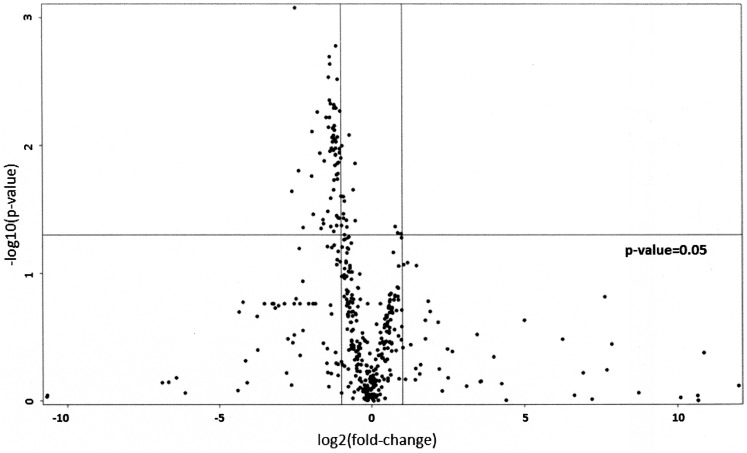
Volcano plot showing differential expressed mi-RNAs between MPM and PAE subjects.

The expression profiles of 754 miRNAs were examined by Openarray, in MPM cases versus PAE subjects taken as controls. Raw data are reported in S1 Table. Openarray data showed that 435 miRNAs were detectable (expressed in at least one sample). In MPM, 62 miRNAs were downregulated with more than a two-fold change in comparison to PAE subjects as shown in Fig 2 (volcano plot).

The top 20 miRNA chosen for validation by a custom RT-qPCR assay according to the previously described criteria are reported in Table 2. 16 out of 20 miRNAs were significantly down-regulated and showed differential expression between the two groups (in bold). The validation step included 22 MPM and 16 PAE subjects: four subjects (1MPM and 3 PAE) were excluded due to the low miRNA yield.

**Table 2:** Validation of the top-20 most significant miRNAs resulting from multiple logistic regression analysis, adjusted for age, sex, BMI and smoking habits.

miRNA name	Fold Change	p-value	FDR
**let-7a**	**0.47**	**0.022**	**0.056**
**miR-103**	**0.25**	**0.012**	**0.056**
**miR-142-3p**	**0.38**	**0.021**	**0.056**
**miR-148b**	**0.29**	**0.013**	**0.056**
**miR-151-5p**	**0.40**	**0.025**	**0.056**
**miR-199a**	**0.32**	**0.016**	**0.056**
**miR-23b**	**0.43**	**0.024**	**0.056**
**miR-27a**	**0.35**	**0.020**	**0.056**
**miR-30e-3p**	**0.37**	**0.014**	**0.056**
**miR-744**	**0.31**	**0.009**	**0.056**
**miR-98**	**0.23**	**0.013**	**0.056**
**let-7f**	**0.41**	**0.03**	**0.063**
**miR-142-5p**	**0.45**	**0.034**	**0.063**
**miR-181a**	**0.47**	**0.037**	**0.063**
**miR-32**	**0.43**	**0.039**	**0.063**
**miR-361**	**0.45**	**0.040**	**0.063**
miR-130a	0.44	0.064	0.085
miR-23a	0.47	0.065	0.085
miR-505	0.48	0.060	0.085
miR-30d	0.48	0.077	0.097

### Discrimination of MPM cases and controls by receiver operating characteristic (ROC) curves

To examine the discrimination ability between cases and controls, we fitted multiple logistic regression models adjusted for sex, age, BMI, and smoking and then calculated adjusted receiver operating characteristic (ROC) curves for each validated miRNA. Table 3 reports the areas under the curve (AUC), 95% CI, sensitivity and specificity values. The same ranking was obtained using AIC and BIC (results not shown).

**Table 3:** Area under the curve (AUC), 95% Confidence Interval, sensitivity and specificity of the 16 validated mi_RNA resulting from Receiving Operating Characteristics (ROC) curve adjusted by age, gender, BMI and smoking).

miRNA	Sensitivity	Specificity	AUC	AUC 95% CI
**miR-103**	**1.000**	**0.667**	**0.864**	**(0.724–1.000)**
**miR-98**	**1.000**	**0.667**	**0.864**	**(0.727–1.000)**
**miR-148b**	**1.000**	**0.733**	**0.852**	**(0.699–1.000)**
**miR-744**	**0.727**	**0.867**	**0.845**	**(0.705–0.986)**
**miR-30e-3p**	**0.636**	**0.933**	**0.827**	**(0.679–0.976)**
miR-199a	0.636	0.933	0.818	(0.675–0.961)
miR-27a	0.727	0.867	0.818	(0.671–0.966)
miR-142-3p	0.682	0.867	0.818	(0.667–0.969)
miR-151-5p	0.682	0.867	0.809	(0.659–0.96)
miR-23b	0.636	0.867	0.791	(0.633–0.949)
miR-181a	0.773	0.733	0.791	(0.632–0.950)
let-7a	0.591	0.933	0.785	(0.626–0.943)
let-7f	0.545	0.933	0.779	(0.616–0.942)
miR-142-5p	0.636	0.867	0.773	(0.608–0.937)
miR-361	0.818	0.667	0.767	(0.601–0.932)
miR-32	0.864	0.600	0.761	(0.599–0.922)

**Fig 3:**
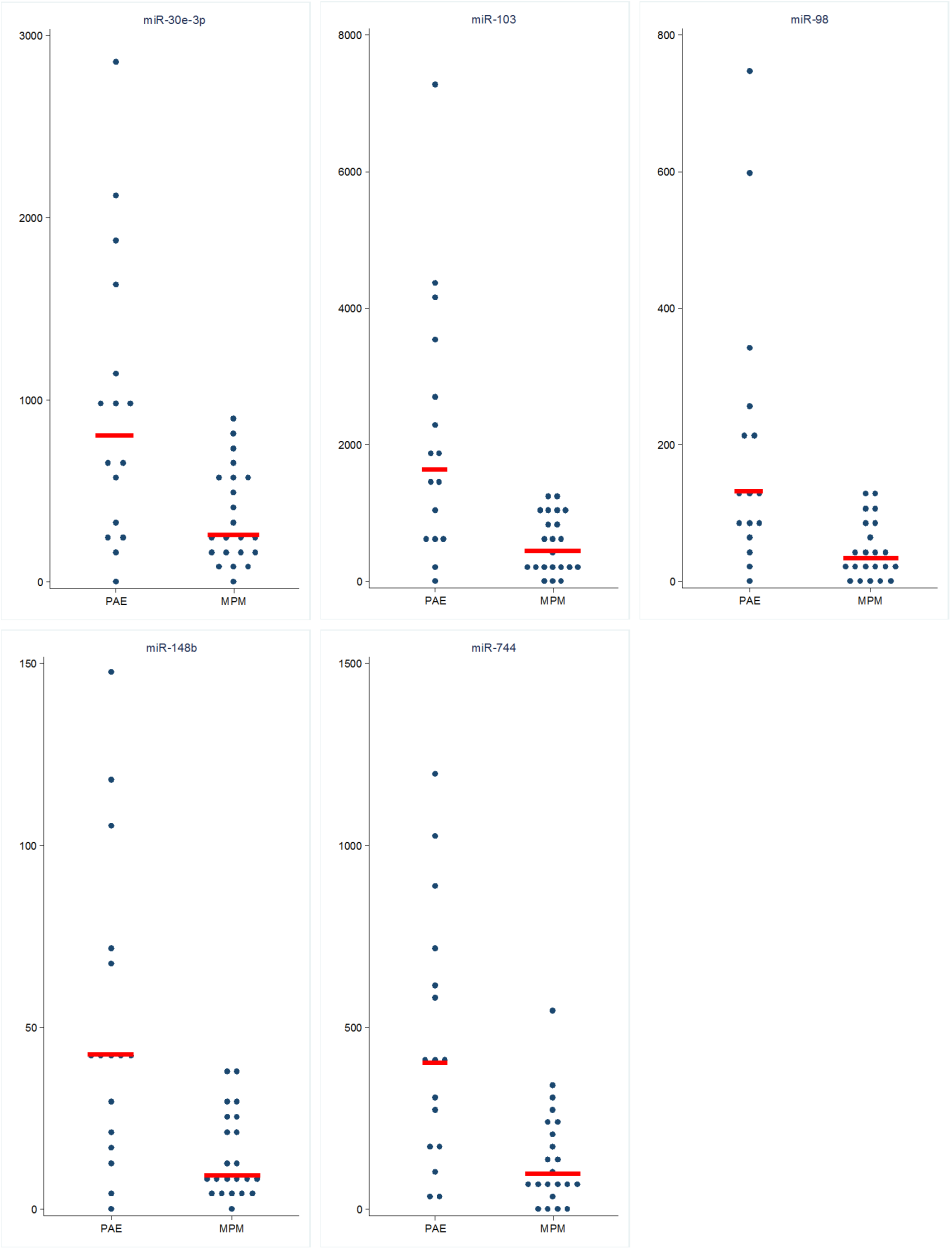
Scatter plot of the 5 best miRNAs in MPM and PAE. Expression values were normalized to RNU48 and expressed as 2 ^-Δcrt^.

The AUC of the 5-best discriminating miRNAs were 0.864 for miR-103a-3p and miR-98, 0.852 for miR-148b, 0.845 for miR-744 and 0.827 for miR-30e-3p. The median value and the interquartile range of these five miRNAs in MPM and PAE subjects are reported in Table 4 and the scatter plot of the relative expression in Fig 3.

**Table 4:** Median value and Interquartile Range if the 5 best mi_RNA in discriminating MPM and PAE subjects.

	MPM cases			PAE subjects			
MiRNA	Median	Lower Quartile	Upper Quartile	Median	Lower Quartile	Upper Quartile	Pvalue *
miR-103	443	185	1028	1631	646	3043	0.0009
miR-30e-3p	257	139	552	800	304	1420	0.001
miR-744	97	57	236	402	182	657	0.001
miR-98	33	11	80	132	67	235	0.0005
miR-148b	9	6	25	42	18	71	0.0045

* Non parametric Wilcoxon rank sum test

All miRNAs were significantly down-regulated in MPM patients. Subgroups comparison by gender, histological types, and smoking habits (current, never, former) did not show significant differences (results not shown).

**Fig 4:**
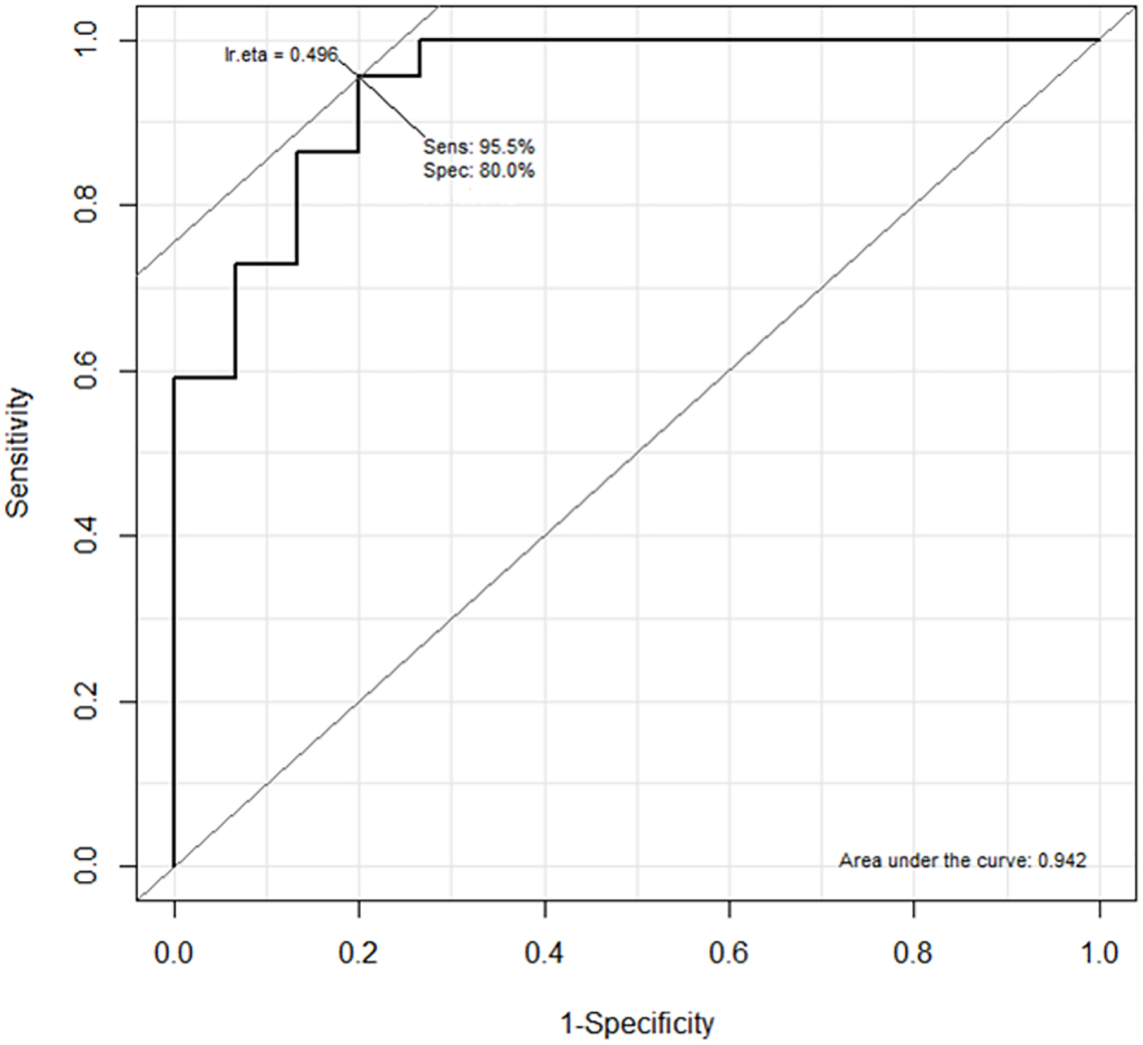
ROC curve of the combination of miR-103a-3p and miR-30e-3p.

When the possible combinations of the 5-best miRNAs were examined, we found that the most discriminating combination was given by miR-103a-3p + miR-98 + miR-30e-3p which generated an AUC of 0.948. MiR-103a-3p and miR98 were highly intercorrelated (r = 0.96, p<0.001). We thus further simplified our signature choosing a more parsimonious model including miR-30e-3p and miR-103a-3p (Likelihood Ratio Test for adding miR-98, p = 0.65) that had been already indicated as a potential diagnostic marker in a similar previous investigation [16]. This signature generated an AUC of 0.942 (95%CI 0.875–1.00) with a Sensitivity of 95.5% and a Specificity of 80.0% (Fig 4). The selected model also showed the best performance in terms of AIC and BIC.

No association was found between miR-103a-3p or miR-30e-3p expression and years of exposure in PAE subjects (p = 0.28 and p = 0.26 respectively).

### Candidate miRNA expression and patient survival

**Fig 5:**
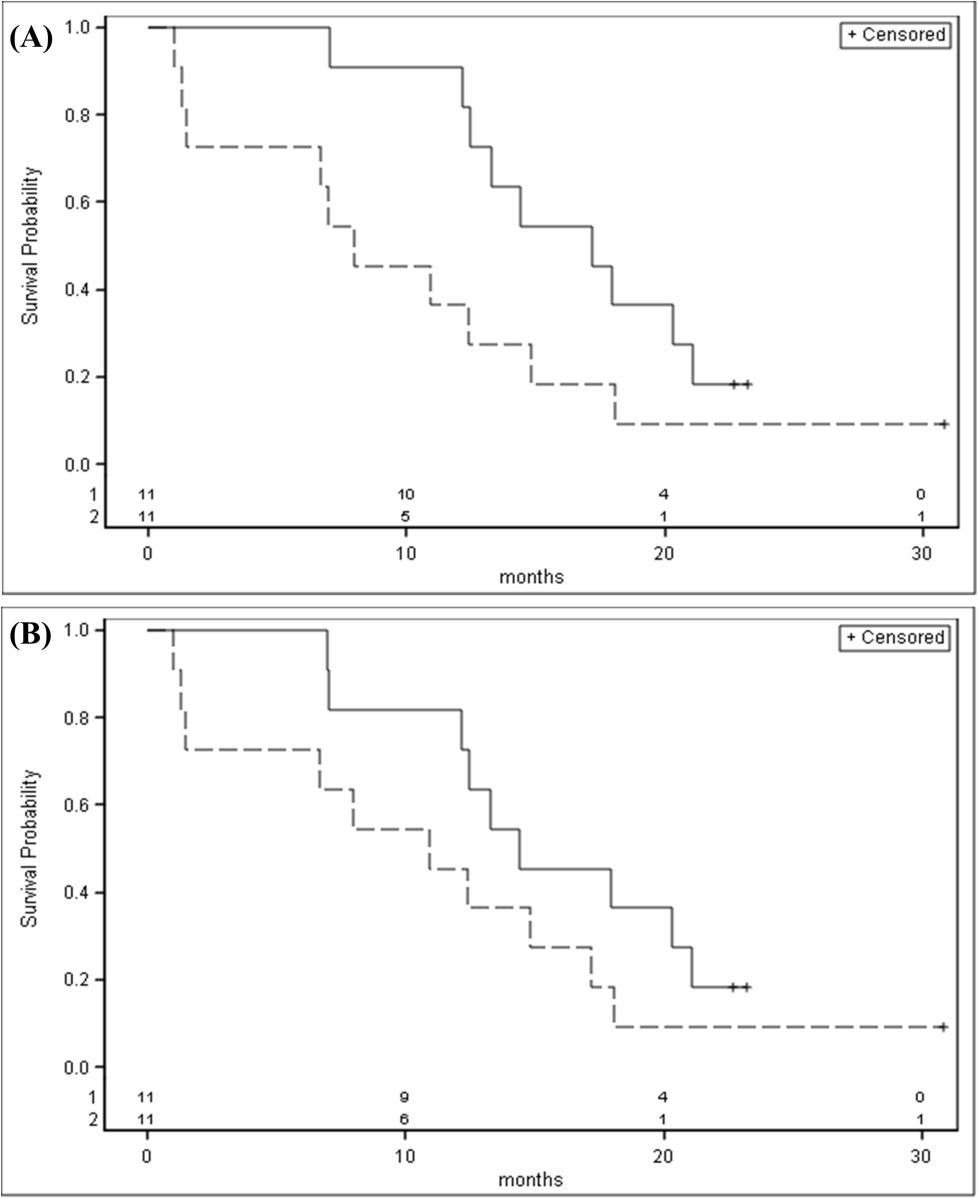
Kaplan-Meier estimate of the overall survival time (months) of MPM stratified for mir-103a-3p (A) and mir-30-e3p (B) expression levels (solid line: above the median; dashed line: under the median).

All but three MPM patients died as of January 2016. The Kaplan–Meier survival analysis showed that patients with miR-103a-3p or miR-30e-3p expression above the median had a better survival than those with expression below the median, although the findings were not statistically significant (p = 0.07 and p = 0.18 respectively). (Fig 5A and 5B).

Using multivariate Cox regression analysis including gender, age, BMI and smoking we found an HR for miR-103a-3p above the median of 0.37 (95%CI 0.13–1.13) and of 0.51 (95%CI 0.17–1.52) for miR-30e-3p.

## Discussion

The aggressive nature, the late diagnosis and the poor prognosis of MPM have pointed out the need of reliable biomarkers able to detect the disease at earlier stages and to improve prognosis. Several studies have shown that miRNA expression profiles can have specific signatures related to both diagnosis and cancer progression [10, 11, 22, 23]. In the last decade, the use of miRNAs as potential biomarkers in MPM has been examined, although most studies analysed miRNAs in tissues or cell cultures [12]. Only a limited number of studies investigated miRNA expression in blood, an easily accessible sample that allows the use of minimally invasive biomarkers.

Santarelli et al. [14] examined serum miR-126 expression in MPM, PAE subjects and healthy controls and found lower levels among cases in comparison to either PAE subjects or healthy controls. The discrimination power among groups was however moderate (Se = 60%, Sp = 74% and Se = 73%, Sp = 74% respectively). In a subsequent study, Tomasetti et al. [15] performed a clinical validation of miR-126 in serum of MPM, lung cancer patients and healthy subjects. Low miR-126 levels were found in both cancer groups when compared to healthy controls. In addition, lowest levels of miR-126 were strongly associated to worse survival.

A miR-625-3p up-regulation was found in plasma from MPM patients compared to healthy controls. Based on ROC analysis, the accuracy of the identified miRNA resulted to be around 0.8. Differential expression of miR-625-3p was also found in tumor samples *vs* non malignant pleura suggesting a potential link between circulating miRNAs and the origin tissue [13].

A serum miRNA signature (including overexpression of miR-25, miR-29 and miR-433) has been recently suggested as associated with MPM histotypes and poor prognosis in a very small number of cases [24].

miR-103a-3p has been shown to be downregulated in MPM patients in comparison to PAE subjects. The AUC was 0.76 with a Se of 83% and Sp of 71% [16]. The combination of miR-103a-3pa-3p and mesothelin improved the biomarker performance with a Se = 95% and Sp = 81% [17].

In this study we examined EV-associated miRNA expression in MPM patients and subjects with past asbestos exposure and found a two miRNA (miR-103a-3p and mir-30-3ep) signature that discriminate the two groups with high accuracy (AUC 0.942), high sensitivity (95.5%) and good specificity (80.0%) thus allowing to avoid false positives in cancer-free subjects and to reduce false negatives among MPM. The lack of association between miRNA expression and duration of asbestos exposure in cancer-free subjects strengthens the role of our signature as potential biomarker to distinguish MPM cases and asbestos exposed subjects.

Although the present study also suggests a better survival in patients characterized by an overexpression of miR-103a-3p or miR-30e-3p, the small sample size prevents any firm conclusion.

Our findings are consistent with previously reported downregulation of miR-103a-3p as potential biomarker for MPM [16, 17]. Other miRNAs recently highlighted in the literature as possible biomarkers of MPM (miR-126, miR-625-3p, miR-25, miR-29 and miR-433) were included in our screening phase and did not significantly differ between cases and controls (see S1 Table).

Analysis based on the combination of miRNA and mesothelin suggested that a multiple-biomarker assay showed superior diagnostic properties than assay based on mesothelin alone or single miRNAs for MPM detection in both general population and asbestos exposed subjects. In the present study we observed a consistently improved diagnostic accuracy obtained from the combination of miR-103a-3p with miR-30-3ep.

The two miRNAs we are proposing as a possible signature of MPM, are known to be involved in cancer development and progression. In particular, miR-103a-3p is reported to be involved in different biological functions, such as cell cycle progression [25] and cell differentiation [26]; while mi-R-30e-3p, a member of the miR 30 family, is involved in the cell growth, apoptosis related to cancer development as it might negatively regulate the expression of Ubc9, a key regulator of the stimulation-mediated cell growth pathway and cancer development [27]. The overall targets of these miRNAs include several genes which are important for cancer development, in particular genes related to the p53 pathway feedback loops [28] making their deregulation a possible important step in the carcinogenic process.

Differences in miRNA expression in different studies can be due to many factors such as control type (asbestos exposed subjects, healthy controls or lung cancer), different methods for measuring miRNA expression, biospecimen type and study size.

In this investigation we used the highly quantitative Open Array technology, which allowed us to analyze, in an unbiased way, 754 miRNAs in one single reaction, producing very precise and accurate data. The candidate miRNAs have been further validated by specific Real time PCR, lowering the possibility of random findings, with a very high success rate (16/20 confirmed miRNAs). Moreover, we conducted miRNA quantification in plasma extracellular vesicles, which are considered as an active mechanism of communication between cancer cells and immune system. Extracellular vesicles have been shown to provide a protective and enriched source of miRNAs for biomarker profiling compared to intracellular and cell-free blood, since they are not diluted amongst other RNA species.

The main limitation of our study is the small number of subjects, as well as lack of subjects with other thoracic diseases (e.g lung cancer, pleural metastatis, benign pleural effusion) that prevents firm conclusions, and underlines the need of larger and prospective studies in order to obtain more reliable data.

In conclusion, our findings suggest an EV-associated miRNA signature able to discriminate MPM from PAE subjects. This signature may be an important non-invasive biomarker for detection of MPM in the future.

## Supporting information

S1 Fig Example of EV quality control. For each batch of plasma analyzed we performed: Transmission electron microscopy (TEM) image of isolated extracellular vesicles from human plasma (Panel A); Flow Cytometry analysis of EVs isolated from human plasma and stained with CFSE (Carboxyfluorescein succinimidyl ester) to assess integrity (Panel B); Size measurement and quantification of EVs by Nanosight (Panel C).
(TIF)

S1 Table Raw data of the screening step. *DB_Screening_miRNAs*: individual miRNA Crt and Amp_score obtained in the screening step (run in single); *DB_Screening_endogenous*: Crt_mean, standard deviation and variation coefficient of the 4 internal controls (including 3 endogenous control RNU48, RNU44 U6 rRNA, and the negative ath-miR control) for each study subject. In both sheets it is also reported whether miRNAs and endogenous controls were included in pool A or B; *DB_Screening_Fold Change*: Fold Change (FC), p-value and FRD of the 435 expressed miRNAs.
(XLSX)

S2 Table Expression data of the top-20 miRNA selected for validation by TLDA and for RNU48 (Ct mean, standard deviation and variation coefficient). (XLSX)
