# Supplementary figures and images for: Plasmatic extracellular vesicle microRNAs in malignant pleural mesothelioma and asbestos-exposed subjects suggest a 2-miRNA signature as potential biomarker of disease
Source: PLoS One. 2017 May 4;12(5):e0176680. doi: 10.1371/journal.pone.0176680 (PMC5417506; doi:10.1371/journal.pone.0176680)

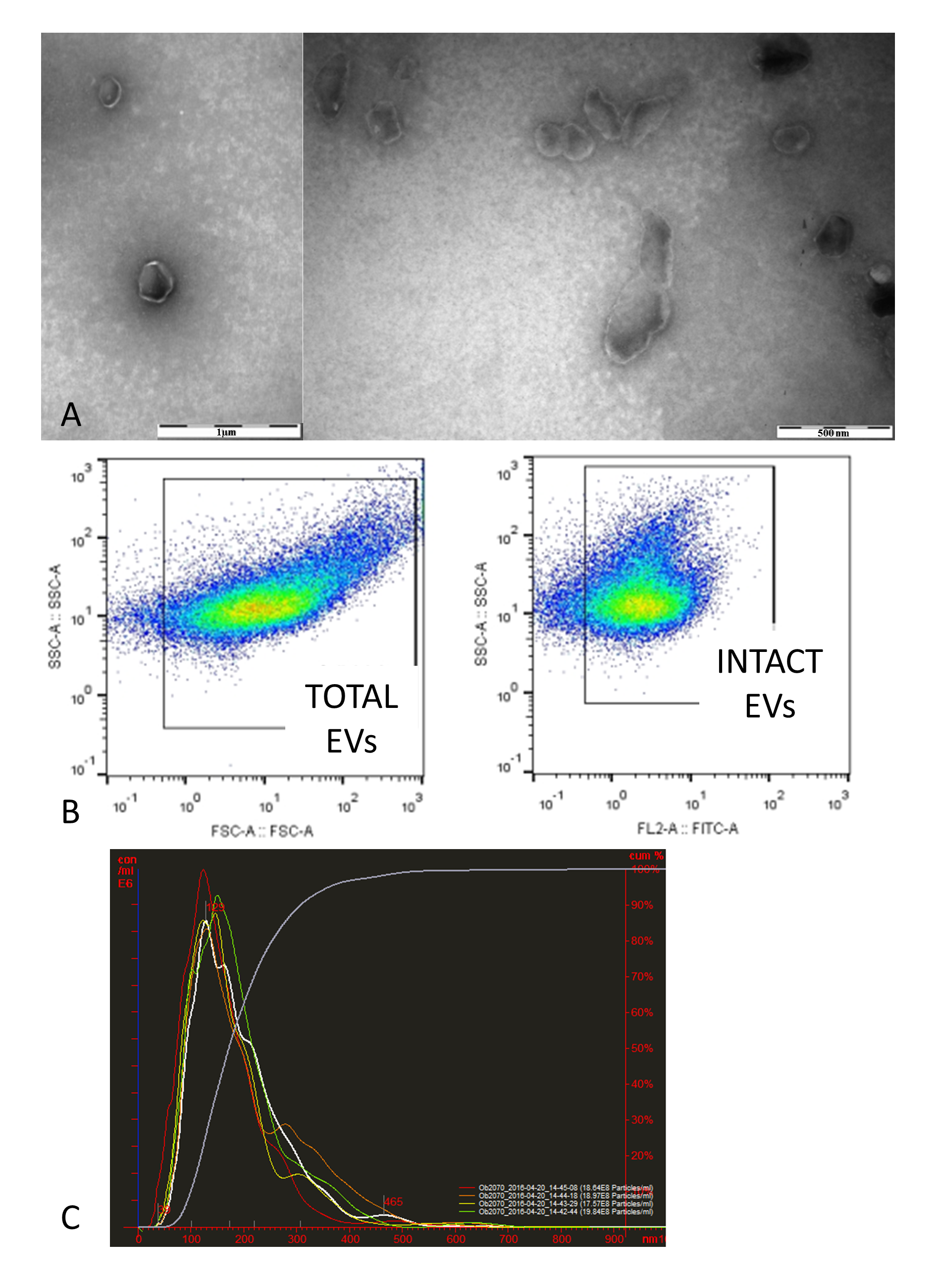

Supplement: S1 Fig — For each batch of plasma analyzed we performed: Transmission electron microscopy (TEM) image of isolated extracellular vesicles from human plasma (Panel A); Flow Cytometry analysis of EVs isolated from human plasma and stained with CFSE (Carboxyfluorescein succinimidyl ester) to assess integrity (Panel B); Size measurement and quantification of EVs by Nanosight (Panel C). (TIF) [file pone.0176680.s001.tif]
